# Performance Evaluation of the Verigene Gram-Positive and Gram-Negative Blood Culture Test for Direct Identification of Bacteria and Their Resistance Determinants from Positive Blood Cultures in Hong Kong

**DOI:** 10.1371/journal.pone.0139728

**Published:** 2015-10-02

**Authors:** Gilman K. H. Siu, Jonathan H. K. Chen, T. K. Ng, Rodney A. Lee, Kitty S. C. Fung, Sabrina W. C. To, Barry K. C. Wong, Sherman Cheung, Ivan W. F. Wong, Marble M. P. Tam, Swing S. W. Lee, W. C. Yam

**Affiliations:** 1 Department of Health technology and Informatics, The Hong Kong Polytechnic University, Hong Kong SAR, China; 2 Department of Microbiology, Queen Mary Hospital, The University of Hong Kong, Hong Kong SAR, China; 3 Department of Pathology, Princess Margaret Hospital, Hong Kong SAR, China; 4 Department of Clinical Pathology, Pamela Youde Nethersole Eastern Hospital, Hong Kong SAR, China; 5 Department of Pathology, United Christian Hospital, Hong Kong SAR, China; University Hospital Münster, GERMANY

## Abstract

**Background:**

A multicenter study was conducted to evaluate the diagnostic performance and the time to identifcation of the Verigene Blood Culture Test, the BC-GP and BC-GN assays, to identify both Gram-positive and Gram-negative bacteria and their drug resistance determinants directly from positive blood cultures collected in Hong Kong.

**Methods and Results:**

A total of 364 blood cultures were prospectively collected from four public hospitals, in which 114 and 250 cultures yielded Gram-positive and Gram-negative bacteria, and were tested with the BC-GP and BC-GN assay respectively. The overall identification agreement for Gram-positive and Gram-negative bacteria were 89.6% and 90.5% in monomicrobial cultures and 62.5% and 53.6% in polymicrobial cultures, respectively. The sensitivities for most genus/species achieved at least 80% except *Enterococcus spp*. (60%), *K*.*oxytoca* (0%), *K*.*pneumoniae* (69.2%), whereas the specificities for all targets ranged from 98.9% to 100%. Of note, 50% (7/14) cultures containing *K*.*pneumoniae* that were missed by the BC-GN assay were subsequently identified as *K*.*variicola*. Approximately 5.5% (20/364) cultures contained non-target organisms, of which *Aeromonas spp*. accounted for 25% and are of particular concern. For drug resistance determination, the Verigene test showed 100% sensitivity for identification of MRSA, VRE and carbapenem resistant *Acinetobacter*, and 84.4% for ESBL-producing Enterobacteriaceae based on the positive detection of *mecA*, *vanA*, *bla*_*OXA*_ and *bla*_*CTXM*_ respectively.

**Conclusion:**

Overall, the Verigene test provided acceptable accuracy for identification of bacteria and resistance markers with a range of turnaround time 40.5 to 99.2 h faster than conventional methods in our region.

## Introduction

Sepsis is the ninth leading cause of mortality in Hong Kong, accounting for nearly 900 deaths in 2014 [1]. It is initially caused by bloodstream infections (BSI) with pathogenic bacteria, due to *Enterobacteriaceae* members and *Staphylococcus spp*. being the predominant etiological agents [2, 3]. The mortality rate was shown to be highly associated with time to effective antimicrobial treatment [3]. The risk of death increased by 6% to 10% for every hour of delay in administration of effective antibiotics [4].

The conventional laboratory diagnosis for BSI relies on broth-based cultures of patients’ blood samples using automated, real-time monitoring blood culture systems. Upon broth positivity, direct Gram stain is performed and a portion of the broth culture is sub-cultivated onto solid media to obtain isolated colonies. The subculture takes 18 to 48-hour incubation prior to biochemical tests for a definitive bacterial identification. With the advent of Matrix-assisted laser desorption ionization-time of flight mass spectrometry (MALDI-TOF MS), the time to identification for blood cultures can be shortened to around 5-hour [5–7]. However, conventional drug susceptibility test is still required to determine the final antimicrobial susceptibility pattern, which normally requires an additional 12–24 hours [8]. The time interval between broth-culture positivity and the availability of final laboratory results leaves the clinicians with limited information to enable clinical decisions during the critical phase of infection. Empiric treatments with broad spectrum antimicrobials are always prescribed to BSI patients in advance of culture and susceptibility results [9]. However in an era of increasing antibiotic resistance, empirical antimicrobial agents may result in suboptimal therapy. In Hong Kong, about 30% of *E*.*coli* isolated from blood culture were positive for extended-spectrum beta-lactamase (ESBL) and more than 50% of patients infected with these resistant strains were actually prescribed with inappropriate empirical treatment [9, 10]. A higher 28-day mortality rate was shown to be significantly associated with ineffective empiric antimicrobial therapies [11]. Beyond its impact on mortality, ineffective treatment is also associated with longer hospital stays [12], which present a significant financial challenge to the health care facility given that hospitalization accounts for more than 80% of the total medical cost used for BSI cases [13]. Rapid microbiological methods that can shorten the turnaround time for bacterial identification and antimicrobial susceptibility tests will definitely facilitate early clinical management, thus reducing the mortality and relieving the corresponding financial burden.

The Verigene Blood Culture Test (Nanosphere, Northbrook, IL) is a closed, fully automated, microarray-based platform that is designed not only for bacterial identification but also drug resistance detection directly from positive blood culture broths within 2.5 hours. The platform consists of two assay panels: the BC-GP assay and the BC-GN assay. The BC-GP assay obtained CE-IVD mark for detection of Gram positive organisms commonly isolated from blood cultures including *Staphylococcus aureus*, *Staphylococcus epidermidis*, *Staphylococcus lugdunensis*, *Staphylococcus spp*., *Micrococcus spp*., *Streptococcus pneumoniae*, *Streptococcus pyogenes*, *Streptococcus agalactiae*, *Streptococcus anginosus* group, *Streptococcus spp*., *Enterococcus faecalis*, *Enterococcus faecium* and *Listeria spp*. In addition, the assay also detects the presence of the *mecA* gene in *S*. *aureus* and *S*. *epidermidis* and *vanA* and *vanB* genes in *E*. *faecalis* and *E*. *faecium*. The BC-GN assay is also a CE-IVD assay designed for the detection of Gram negative bacteria that are frequently associated with sepsis, which includes *Acinetobacter spp*., *Citrobacter spp*., *Enterobacter spp*., *Escherichia coli*, *Klebsiella pneumoniae*, *Klebsiella oxytoca*, *Proteus spp*., *Pseudomonas aeruginosa* and *Serratia marcescens*, as well as the drug resistance genes encoding for β-lactamases and carbapenemases–*bla*_*CTX-M*_, *bla*_*IMP*_, *bla*_*VIM*_, *bla*_*KPC*_, *bla*_*NDM*_, and *bla*_*OXA*_.

Evaluation studies for the Verigene test have been extensively conducted in the U.S. and Europe to assess the diagnostic performance. The sensitivity and specificity of the assay for bacterial identification ranged from 92.6%–98.6% and 95.4%–99.5% respectively. The concordant rate of resistance detection ranged from 88.9% to 100% as compared to phenotypic susceptibility tests [14–20].

However, it should be noted that the performance reported in Western countries might not be reproducible in Hong Kong given that the bacterial lineages and drug resistance patterns frequently encountered in our region are not congruent with the spectra circulating in the U.S. and Europe. This can be exemplified by the observation that Gram positive bacteria accounted for 52% to 77% of bacterial sepsis in the U.S. whereas Gram negative bacilli, particularly *E*.*coli*, represented causative agents for about 60% of BSI cases in Hong Kong [21–23]. Therefore, regional evaluation is necessary to determine the applicability of the assay in our hospitals.

In this study, we investigated the diagnostic accuracy (sensitivity and specificity) of the Verigene BC-GP ad BC-GN assays to identify bacteria and their resistance determinants directly from positive blood culture broths collected from four public hospitals serving different district areas in Hong Kong. A retrospective comparison of time to identification between the Verigene Blood Culture Test and conventional culture-based methods was also conducted at one of the study sites.

## Materials and Methods

### Collection of Blood Culture Broths

A total of 364 positive blood culture broths from non-duplicated patients with suspected sepsis were collected from January 2014 to May 2014 at four public acute hospitals located at different district areas across Hong Kong, namely 1,633 bed Pamela Youde Nethersole Eastern Hospital (PYNEH), 1,753 bed Princess Margaret Hospital (PMH), 1,403 bed United Christian Hospital (UCH) and 1,702 bed Queen Mary Hospital (QMH). Bactec plus/F (Becton Dickson, US) aerobic culture bottles were used in all the study sites except for PMH where the BacT/Alert FA (bioMerieux, France) system was used. The bottles were incubated in the corresponding automated blood culture system until positive within five days. Upon broth positivity, samples from PYNEH, PMH and UCH were sent to The Hong Kong Polytechnic University for the Verigene Blood Culture Test within 24 hours. If the samples could not be run within 24 hours, they were stored at 4°C for up to 48 hours. For QMH, after flagging positive by the blood culture system, the samples were almost processed immediately with BC-GP or BC-GN tests using another Verigene system established in their laboratory.

### Conventional Culture-Based Methods for Bacterial Identification and Drug Susceptibility Test

Positive blood culture broths were inoculated onto Columbia blood agar, chocolate agar and MacConkey agar for overnight incubation at 37°C in the presence of 5% CO_2_. The isolated colony(s) were identified by automated biochemical platform, Vitek 2 (bioMérieux). Kirby-Bauer disk diffusion method were performed and interpreted according to CLSI guideline (M100-S21) to determine the drug susceptibilities [8]. Further tests were done to confirm the presence of methicillin resistant *S*. *aureus* (MRSA), vancomycin resistant *Enterococci* (VRE), ESBL-producing *Enterobacteriaceae* and carbapenem resistant *Enterobacteriaceae* (CRE) [8]. MRSA was confirmed by resistance to cefoxitin disk and positive growth of *S*. *aureus* colonies (mauve in colour) on the ChromID MRSA agar (bioMérieux) whereas VRE was primarily detected by brain heart infusion agar supplemented with 6μg/mL vancomycin, followed by confirmation with E-test (bioMérieux). Combined disk method (CDM) using ceftazidime with and without clavulanic acid was used as a confirmatory test for ESBL in *E*.*coli*, *Klebsiella spp*., and *Proteus spp*. For *Enterobacter spp*., *Citrobacter spp*. and *Serratia marcescens*, cefepime disk and cefepime-clavulanic acid disk were used for detection of ESBL [24]. For CRE determination, *Enterobacteriaceae* with zone diameter <23mm for imipenem were subjected to combined disk methods using ertapenem, meropenem and imipenem disks placed adjacent to aminophenyl boronic acid (APBA) and ethylenediaminetetraacetic acid (EDTA) as the phenotypic confirmatory test. To avoid bias, technologists conducting the conventional tests were blinded to the results obtained using the Verigene test.

### Verigene BC-GP and BC-GN Assay

Based on the primary Gram stain results, cultures with Gram positive organisms were subjected to the Verigene BC-GP assay whereas cultures containing Gram negative organisms were tested with the Verigene BC-GN assay. All the procedures were carried out according to manufacturer’s instructions. In brief, a total of 350μl and 700μl of positive blood cultures were loaded into the corresponding extraction trays of the BC-GP assay and the BC-GN assay respectively. The extraction trays were then transferred to the processor SP along with other consumables and the test cartridge containing the microarray slides. The instrument automatically performed nucleic acid extraction, purification, microarray hybridization and signal amplification. The whole process took 2.35 hours for the BC-GP assay or 1.88 hour for the BC-GN assay. Upon completion, the test cartridge was removed from processor SP. The microarray slide was manually inserted into Verigene Reader for analysis. The light source of the Verigene Reader excited the signal-enhanced nanoparticles that were specifically bound to the bacteria-specific or resistance-gene-specific probes captured on the slide. The relative brightness of each spot were measured by the photosensor. Positive and negative results were reported as “Detected” or “Not detected” for each bacteria and resistance determinants featured by the Verigene test respectively. “No Call” flag was given to the samples with indeterminate results. These samples were repeated a single time. The repeated result was considered as the final result for the sample.

### Discrepant Analysis for Bacterial Identification and Drug Resistance

Regarding the bacterial identification, isolates generating discrepancies between the conventional culture-based method and the Verigene test were analyzed by 16s ribosomal RNA (rRNA) sequencing according to previous studies [25, 26]. The sequence similarity was determined using the EzTaxon server (http://eztaxon-e.ezbiocloud.net/ezt_identify). *yggE* gene sequencing was performed to differentiate *K*. *variicola* from *K*. *pneumoniae* [27].

In case of discrepancies between genotypic drug resistance results inferred by the Verigene test and phenotypic susceptibility test results, PCR-based assays were performed to confirm the presence of resistance genes.

For discordance in methicillin resistance in the specimens with *S*. *aureus* and *S*. *epidermidis*, our in-house real time polymerase chain reaction (PCR) assay were used to detect the presence of *mecA* gene [28]. In case of contradictory VRE results, the presence of *vanA* and *vanB* genes was verified by vanA- and vanB-specific PCR assays according to the protocol described by Bell *et al [29].* For the mismatches in susceptibilities to β-lactam drugs in Gram negative organisms, the presence of resistance genes associated with β-lactamases, such as *bla*_*TEM*_, *bla*_*SHV*_, *bla*_*CTXM*_, *bla*_*OXA*_, *bla*_*KPC*_ and *bla*_*NDM*_ were firstly identified by multiplex PCR assays [30, 31] followed by genotyping using bi-directional Sanger sequencing.

### Assessment of Time to Identification Using the Verigene Test and Conventional Culture-Based Methods

A retrospective comparison of turnaround time for bacterial identification was conducted using a total of 125 positive blood cultures collected from QMH, in which 38 cultures were positive for Gram positive organisms whereas 87 cultures contained Gram negative organisms. These cultures were a subset of the 364 cultures collected for evaluation of the diagnostic performance of the Verigene test and all of them were positive for the organisms featured by the Verigene panel. Unlike the other three study sites, QMH had three Verigene systems established in their clinical microbiology laboratory, which allowed more accurate comparison of time to result between the Verigene test and routine culture-based methods. The time at which the primary Gram stain result was reported was considered as time-zero for turnaround time determination. For the Verigene test, the time elapsed between time-zero and the completion time of the BC-GP assay and the BC-GN assay recorded in the Verigene system was considered as the time to result. For the conventional culture-based method, the time at which final results were input into the laboratory information system by technologists was used to determine the total time required for bacterial identification.

### Data Processing and Statistical Analysis

The diagnostic sensitivity and specificity of the Verigene BC-GP and BC-GN assays for bacterial identification and drug resistance determination were calculated using standard methods. Ninety-five percent confident intervals (95% CI) were calculated using the adjusted Wald method by free software available from http://www.measuringusability.com/wald.htm. Pair *t-*test or Wilcoxon signed rank test where appropriate was used to determine the statistically significant difference in time to result between the Verigene tests and the routine conventional methods.

### Ethical Considerations

The study protocol was carefully reviewed and approved by the Human Subjects Ethics Sub-committee under the University Research Committee of The Hong Kong Polytechnic University. Individual informed consent was waived by the ethics committee because this study used currently existing samples collected during the course of routine medical care and did not pose any additional risks to the patients.

The Verigene Blood Culture Test are not currently approved for standard clinical procedure by Hospital Authority in Hong Kong and not ethically permitted for clinical diagnosis. Thus, we did not inform the clinicians of the results obtained from the methods.

## Results

### Overall Concordance

Among the 364 blood culture samples collected in the study, 78,8% (287/364) and 94.2% (343/364) were flagged positive in the blood culture system within 24-hour and 48-hour incubation respectively. Overall, 31.3% (n = 114) and 68.7% (n = 250) were found to contain Gram positive and Gram negative bacteria respectively on the primary Gram stain. A total of 16 cultures (11 Gram Positive and 5 Gram negative) generated an indeterminate result in the initial analysis, giving the initial call rate of 95.6%. Of these, two were resolved following a single retest for a final call rate of 96.2% (350/364). In this study, only 5.5% (20/364) cultures contained organisms not included on the Verigene test panel. Among 364 blood cultures, 90.1% (n = 328) were monomicrobial, and 9.9% (n = 36) contained at least two bacteria. The overall concordance rate in bacterial identification between the Verigene test and reference culture method was 86.8% (BC-GP: 87.7%; BC-GN: 86.4%). Higher concordance was obtained in monomicrobial cultures (90.2%) than in polymicrobial cultures, of which only 55.6% (20/36) showed fully concordant identification. The performances of the Verigene test in each study site are presented in Table 1. Of all the collected blood cultures, 94.0% (342/364) were tested with the Verigene test within 24 hour after flagging positive by the blood culture system as suggested by the manufacturer. For the 22 samples that were not analyzed within this period, they were stored at 4°C up to 48 hours. The Verigene test results obtained for these samples (BC-GP: n = 4; BC-GN: n = 18) were all concordant with reference culture method, indicating that the period of specimen storage prior to the Verigene test did not affect the test results in this study.

**Table 1:** The overall concordance in bacterial identification between Verigene test and culture-based method in different study sites.

	BC-GP Test			BC-GN Test			Combined		
Study Site	No. of tested sample	No. of sample concordant with culture-based method	Concordance rate % (95% CI^a^)	No. of cultures	No. of sample concordant with culture-based method	Concordance rate % (95% CI^a^)	No. of cultures	No. of sample concordant with culture-based method	Concordance rate (95% CI^a^)
**Monomicrobial cultures**									
QMH	43	36	83.7 (69.7–92.2)	96	80	83.3 (74.5–89.6)	139	116	83.5 (76.3–88.8)
PMH	28	24	85.7 (67.9–94.9)	51	48	94.1 (83.5–98.6)	79	72	91.1 (82.6–95.9)
UCH	20	20	100 (85.9–100)	40	38	95.0 (82.6–99.5)	60	58	96.7 (88.0–99.8)
PYNEH	15	15	100 (82.0–100)	35	35	100 (91.4–100)	50	50	100 (93.9–100)
All Sites	106	95	89.6 (82.2–94.3)	222	201	90.5 (85.9–93.8)	328	296	90.2 (86.5–93.0)
**Polymicrobial cultures**									
QMH	5	4	80.0 (36.0–98.0)	14	5	35.7 (16.2–61.4)	19	9	47.4 (27.3–68.3)
PMH	-	-	-	4	3	75.0 (28.9–96.6)	4	3	75.0 (28.9–96.6)
UCH	-	-	-	3	2	66.7 (20.2–94.4)	3	2	66.7 (20.2–94.4)
PYNEH	3	1	33.3 (5.6–79.8)	7	5	71.4 (35.2–92.4)	10	6	60.0 (31.2–83.3)
All Sites	8	5	62.5 (30.4–86.5)	28	15	53.6 (35.8–70.5)	36	20	55.6 (39.6–70.5)
**Overall**	**114**	**100**	**87.7 (80.3–92.7)**	**250**	**216**	**86.4 (81.6–90.1)**	**364**	**316**	**86.8 (82.9–89.9)**

QMH: Queen Mary Hospital; PMH: Princess Margaret Hospital; UCH: United Christian Hospital; PYNEH: Pamela Youde Nethersole Eastern Hospital; 95%CI: 95% confidence interval

^a^ 95% confidence interval was calculated using Adjusted Wald Method (http://www.measuringu.com/wald.htm)

### Bacterial Identification for Gram Positive Organisms

From 114 blood cultures containing Gram positive organisms, a total of 121 isolates were obtained (Table 2). The majority of these belonged to *Staphylococcus spp*, which accounted for 60.3% (73/121) of all Gram positive organisms isolated in this study, followed by *Streptococcus spp*., 22.3% (27/121), *Enterococcus spp*., 9.9% (12/121), and *Listeria spp*., 0.8% (1/121). A total of 8 isolates were a genus/species not included in BC-GP panel, including *Bacillus spp*. (n = 3), *Kocuria spp*. (n = 3), *Corynebacterium spp*. (n = 1) and *Candida krusei* (n = 1) (Table 2).

**Table 2:** Performance of BC-GP for identification of Gram positive organisms.

	No. (%) of isolates								
Organisms	Total	Correctly identified	Not detected	Misidentified	No Call	Sensitivity (%)	95% CI^a^	Specificity (%)	95% CI^a^
*Staphylococcus spp*.	73 (60.3)	69 (94.5)	-	-	4	100	95.5–100	100	93.6–100
*S*. *aureus*	48 (39.7)	47 (97.9)	-	-	1	100	93.5–100	100	95.7–100
*S*. *epidermidis*	4 (3.3)	4 (100)	-	-	-	100	54.3–100	100	97.3–100
*Streptococcus spp*.	27 (22.3)	25 (92.6)	-	-	2	100	88.4–100	98.9	92.1–99.9
*S*. *pyogenes*	2 (1.7)	2 (100)	-	-	-	100	36.9–100	100	97.3–100
*S*. *agalactiae*	3 (2.5)	3 (100)	-	-	-	100	47.0–100	100	97.3–100
*S*. *anginosus gr*.	1 (0.8)	1 (100)	-	-	-	100	22.4–100	100	97.4–100
*S*. *pneumoniae*	7 (5.8)	7 (100)	-	-	-	100	67.8–100	99.1	94.7–99.9
*E*. *faecalis*	7 (5.8)	4 (57.1)	2 (28.6)	-	1	66.7	29.6–90.8	100	97.2–100
*E*. *faecium*	5 (4.1)	2 (40)	2 (40)	-	1	50	15.0–85.0	100	97.3–100
*Listeria*	1 (0.8)	1 (100)	-	-	-	100	22.4–100	100	97.4–100
Nontarget	8 (6.6)	-	4 (50)	1	3				
*Bacillus spp*.	3	-	2	-	1				
*Candida krusei*	1	-	1	-	-				
*Diphtheroids*	1	-	1	-	-				
*Kocuria spp*.	3	-	-	1^b^	2				
**Total**	**121**	**101**	**8**	**1**	**11**				

^*a*^ *95% confidence interval was calculated using Adjusted Wald Method (* *http*:*//www*.*measuringu*.*com/wald*.*htm*)

^b^ Misidentifed as *S*. *pneumoniae*

Among 73 cultures containing *Staphylococcus spp*., four monomicrobial cultures, including one harbouring *S*. *aureus* and three containing *S*. *haemolyticus*, were reported as “No Call” in spite of single retest (Table 3). For those which had valid verigene results, *Staphylococcus spp*. were identified with 100% (69/69) sensitivity and 100% (48/48) specificity. *S*. *aureus* represented the most predominant species of Gram positive organisms isolated in this study, with sensitivity and specificity of 100%. BC-GP test also identified all *S*. *epidermidis* isolates (n = 4) with no false positivity (Table 2). Other CoNS isolated in this study included *S*. *hominis* (n = 8), *S*. *caprae* (n = 4), *S*. *intermedius* (n = 3), and *S*. *jettensis* (n = 3). All of them were correctly reported as *Staphylococcus spp*. in the BC-GP test.

**Table 3:** The identities of bacteria in cultures with discordant results reported by culture-based method and the Verigene Test.

Culture results	No. of culture	Verigene Test results	16s Sequencing (Sequence similarity %)
Monomicrobial Culture			
*S*. *aureus*	1	No Call	*S*. *aureus subsp*. *aureus* (100%)
CNS	3	No Call	*S*. *haemolyticus* (99.7%)
*S*. *viridans*	1	No Call	*S*. *mitis* (99.27%)
*S*.*gallolyticus*	1	No Call	*S*. *gallolyticus* subsp. pasteurianus (100%)
*E*. *faecalis*	1	No Call	*E*. *faecalis (100%)*
*E*. *faecium*	2	Not Detected	*E*. *faecium (100%)*
*E*. *faecium*	1	No Call	*E*. *faecium (100%)*
*Kocuria spp*.^*a*^	1	*S*. *pneumoniae*	*Kocuria koreensis (97*.*28%)*
*E*. *coli*	2	Not Detected	*E*. *coli (100%)*
*K*. *pneumoniae*	1	Not Detected	*K*. *pneumoniae subsp*. *pneumoniae (99*.*83%)*
*K*. *pneumoniae*	7	Not Detected	*K*. *variicola (100%)*
*K*. *pneumoniae*	1	*K*. *pneumoniae / K*. *oxytoca*	*K*. *pneumoniae subsp*. *ozaenae (100%)*
*K*. *pneumoniae*	1	*K*. *pneumoniae / Enterobacter spp*.	*K*. *pneumoniae subsp*. *ozaenae (100%)*
*P*. *aeruginosa*	2	Not Detected	*P*. *aeruginosa (100%)*
*K*. *oxytoca*	3	Not Detected	*K*. *oxytoca(100%)*
*R*. *planticola* ^*a*^	1	*K*. *oxytoca*	*R*. *planticola (100%)*
**Polymicrobial Culture**			
*CNS / E*. *faecalis*	1	*Staph*. *Spp*	*S*. *petrasii subsp*. *petrasii (100%) / E*. *faecalis (100%)*
*S*. *aureus / E*. *faecalis*	1	*S*. *aureus*	*S*. *aureus subsp*. *aureus (100%) / E*. *faecalis (100%)*
*E*. *coli / P*. *aeruginosa*	1	*P*. *aeruginosa*	*E*. *coli (100%) / P*. *aeruginosa (100%)*
*E*. *coli / P*. *aeruginosa*	1	*E*.*coli*	*E*. *coli (100%) / P*. *aeruginosa (100%)*
*E*. *coli / Proteus*	1	*Proteus spp*.	E.coli (99.86%) / Proteus mirabilis (100%)
*E*. *coli / K*. *pneumoniae*	1	*K*. *pneumoniae / Enterobacter spp*.	*E*. *coli (100%) / K*. *pneumoniae subsp*. *pneumoniae (99*.*85%)*
*E*. *coli/ K*. *pneumoniae*	6	*E*.*coli*	*E*. *coli (100%) / K*. *pneumoniae subsp*. *pneumoniae (99*.*85%)*
*P*. *mirabilis / M*. *morganii* ^*a*^	1	No Call	*P*. *mirabilis* (100%) / *M*. *morganii* (100%)
*E*. *coli / E*.*coli / P*.*mirabilis / K*. *pneumoniae*	1	*Proteus spp*. */ K*.*pneumoniae*	*E*. *coli (100%) / E*. *coli (100%) / P*. *mirabilis (100%) / K*. *pneumoniae subsp*. *pneumoniae (99*.*83%)*

^a^ Organisms are not included in the Verigene Test identification panel

For *Streptococcus spp*., all the species-level targets, namely *S*. *pyogenes* (n = 2), *S*. *agalactiae* (n = 3), *S*. *anginosus* group (n = 1) and *S*. *pneumoniae* (n = 7) yielded 100% sensitivity. A total of 14 *Streptococcus spp*. not included in the BC-GP panel were isolated, including *S*. *mitis* (n = 6), *S*. *dysgalactiae* (n = 3), *S*. *bovis* (n = 3), *S*. *gallolyticus* (n = 1) and *S*. *sanguinis* (n = 1). All were identified as *Streptococcus spp*. by BC-GP test except two (one *S*. *mitis* monomicrobial culture and one *S*. *gallolyticus* monomicrobial culture) were reported as “No Call” (Table 3). With the exception of one monomicrobial culture of *Kocuria spp*. falsely identified as *S*. *pneumoniae*, all the species-level targets for *Streptococci* yield 100% specificity (Table 2).

Compared with *Staphylococcus spp*. and *Streptococcus spp*., the accuracy of the BC-GP for *Enterococcus spp*. was much lower. The sensitivities for *E*. *faecalis* and *E*. *faecium* achieved only 66.7% (4/6) and 50% (2/4) respectively (Table 2). Four cultures (two *E*. *faecalis* and two *E*. *faecium*) had results of “Not Detected”, and thus were considered as false negative cases. Additionally, invalid results (No Call) were obtained for two monomicrobial cultures containing *E*. *faecalis* and *E*. *faecium* respectively (Table 3). Only one blood culture harbouring *L*. *monocytogenes* was obtained in this study. The BC-GP was 100% sensitive and specific for the identification of this organism. For the eight nontarget organisms, 50% of them were correctly assigned as “Not Detected” in the BC-GP Test. One monomicrobial culture with *Bacillus spp*. and one mixed culture of two *Kocuria spp*. isolates yielded invalid results, and one *Kocuria rosea* isolate was misidentified as *S*. *pneumoniae* in the BC-GP test.

### Bacterial Identification for Gram Negative Organisms

A total of 279 Gram negative bacteria were isolated, in which *E*. *coli* was the most frequently isolated species, accounting for 59.1% (165/279) of Gram negative bacteria and 41.3% (165/400) of total bacteria isolated in this study. *K*. *pneumonia* ranked the second, 18.6% (52/279), followed by *P*. *aeruginosa*, 6.1% (17/279), *Proteus spp*., 3.6% (10/279), *Enterobacter spp*., 3.6% (10/279), *Acinetobacter spp*., 1.4% (4/279), *K*. *oxytoca*, 1.1% (3/279), *Citrobacter spp*., 0.4% (1/279), and *Serratia marcescens*, 0.4% (1/279) (Table 4).

**Table 4:** Performance of BC-GN for idenifcation of Gram negative organisms.

	No. (%) of isolates								
Organisms	Total	Correctly identified	Not detected	Misidentified	No Call	Sensitivity (%)	95% CI^a^	Specificity (%)	95% CI^a^
*E*.*coli*	165 (59.1)	158 (95.8)	6 (3.6)	1^c^	-	95.8	91.3–98.1	100	97.2–100
*K*. *pneumoniae*	52 (18.6)	36 (69.2)	14 (27)^d^	2^e^	-	69.2^d^	55.7–80.2	100	98.6–100
*P*. *aeruginosa*	17 (6.1)	13 (76.5)	3 (17.6)	-	1	81.3	56.2–94.2	100	98.8–100
*Proteus spp*.	10 (3.6)	9 (90)	-	-	1	100	73.1–100	100	98.8–100
*Enterobacter spp*.	10 (3.6)	8 (80)	-	-	2	100	70.7–100	99.3	97.2–99.9
*Acinetobacter spp*.	4 (1.4)	4 (100)	-	-	-	100	54.3–100	100	98.8–100
*K*. *oxytoca*	3 (1.1)	-	3 (100)	-	-	0	0.0–53.0	99.3	97.2–99.9
*Citrobacter spp*.	1 (0.4)	1 (100)	-	-	-	100	22.4–100	100	98.8–100
*Serratia marcescens*	1 (0.4)	1 (100)	-	-	-	100	22.4–100	100	98.8–100
Nontarget^b^	16 (5.7)	-	14 (87.5)	1	1				
*Aeromonas veronii*	5	-	5	-	-				
*Morganella morganii*	3	-	2	-	1				
*Salmonella spp*.	2	-	2	-	-				
*Alcaligenes faecalis*	1	-	1	-	-				
*Burkholderia pseudomallei*	1	-	1	-	-				
*Haemophilus influenzae*	1	-	1	-	-				
*Raoultella planticola*	1	-	-	1^f^	-				
*Pseudomonas putida*	1	-	1	-	-				
*Stenotrophomonas maltophilia*	1	-	1	-	-				
***Total***	**279**	**0**	**40**	**4**	**5**	** **	** **	** **	** **

^a^95% confidence interval was calculated using Adjusted Wald Method (http://www.measuringu.com/wald.htm)

^b^ Species were confirmed by 16s rRNA sequencing

^c^ Misidentified as *Enterobacter spp*. by BC-GN

^d^ Seven isolates were confirmed to be *K*. *variicola* instead of *K*. *pneumoniae*by 16s rRNA and yGGEsequencing. After the discrepancy resolution, the sensitivity for *K*. *pneumoniae* was raised to 80% (36/45).

^e^ One was misidentified as mixed culture of *K*. *pneumoniae* and *K*. *oxytoca* whereas the other one was misidentified as mixed cultured of *K*. *pneumoniae* and *Enterobacter*

^f^ Misidentified as *K*. *oxytoca*

The sensitivities of the BC-GN test for each genus/species were as follow: *Acinetobacter spp*., *Citrobacter spp*., *Enterobacter spp*., *Proteus spp*. and *Serratia marcescens*: 100%; *E*.*coli*: 95.8%; *P*. *aeruginosa*: 81.3%; *K*. *pneumoniae*: 69.2%; and *K*. *oxytoca*: 0% (Table 4).

Of the 26 isolates with false negative results by the BC-GN test, 53.8% (14/26) were identified as *K*. *pneumoniae* by Vitek 2. However, 16s rRNA sequencing of these isolates revealed that 50% (7/14) of them indeed shared 100% similarity to *Klebsiella variicola* (Table 3). As confirmed by *yggE* gene sequencing, *K*. *variicola* was considered as the final identification for these seven monomicrobial cultures, and sensitivity for *K*. *pneumoniae* was raised to 80% (36/45) after the discrepancy resolution. Among the 6 isolates reported as *E*. *coli* by both Vitek 2 and 16s rRNA sequencing but not detected by the BC-GN assay, two were obtained from monomicrobial cultures and four were isolated from polymicrobial bacteremia cases including one bimicrobial culture with *E*. *coli* and *P*. *aeruginosa*, one bimicrobial with *E*.*coli* and *P*. *mirabilis*, and one tetramicrobial culture containing *K*. *pneumoniae*, *P*. *mirabilis* and two *E*. *coli* strains (Table 3). In addition, the BC-GN assay reported false negative results for three cultures containing *P*. *aeruginosa*, including two monomicrobial cultures and one mixed culture with *E*. *coli*. The 16s rRNA sequences of these three isolates showed 100% similarity to *P*. *aeruginosa*. Surprisingly, the BC-GN assay failed to detect all the three monomicrobial culture of *K*. *oxytoca* as confirmed by Vitek 2 and 16s rRNA sequencing.

All the targets featured by the BC-GN panel yielded 100% specificity except for *Enterobacter spp*. (99.3%) and *K*. *oxytoca* (99.3%). One mixed culture of *E*. *coli* and *K*. *pneumoniae* was wrongly reported as *Enterobacter spp*. and *K*. *pneumoniae*, whereas two monomicrobial cultures of *K*. *pneumoniae* misidentified as *K*. *pneumoniae* / *K*. *oxytoca* and *K*. *pneumoniae* / *Enterobacter spp*. respectively (Tables 3 and 4).

A total of 16 Gram negative bacteria isolated in this study were not included in the BC-GN panel, namely *Aeromonas hydrophila* (n = 5), *Morganella morganii* (n = 3), *Salmonella spp*. (n = 2), *Alcaligenes faecalis* (n = 1), *Burkholderia pseudomallei* (n = 1), *Haemophilus influenza* (n = 1), *Raoultella planticola* (n = 1), *Pseudomonas putida* (n = 1), and *Stenotrophomonas maltophilia* (n = 1). Fourteen of them were corrected reported as “Not Detected” by the BC-GN test. One monomicrobial culture of *R*. *planticola* was misidentified as *K*. *oxytoca*, whereas one mixed culture containing *P*. *mirabilis* and *M*. *morganii* were given an invalid result (Table 3).

### Identification of Drug Resistance Determinants

With the exception of one “No Call” result obtained for a monomicrobial culture containing MRSA, the BC-GP assay correctly identified all (26/26) MRSA and (4/4) MRSE with no false positivity when compared with culture-based methods (Table 5). Vancomycin resistance was observed in only one *E*. *faecium* isolate out of 12 *Enterococcus spp*. recovered from positive blood cultures as determined by vancomycin E-test method. The BC-GP assay accurately reported a *vanA* positive result for this VRE isolate (Table 5).

**Table 5:** Performance of Verigene Test in detection of drug resistant organisms.

	No. of isolates							
Drug resistant Organisms	Total	Correctly Detected	Not Detected	No Call	Sensitivity (%)	95% CI^a^	Specificity (%)	95% CI^a^
**Gram Positive**								
MRSA	27	26	0	1	100	88.8–100	100	96.6–100
MRSE	4	4	0	0	100	54.3–100	100	97.3–100
VRE	1	1	0	0	100	22.4–100	100	97.4–100
**Gram Negative**								
Cefotaxime resistant *Enterobacteriaceae* (including ESBL producers)	61	38	22	1	63.3	50.7–74.4	100	98.5–100
ESBL producing *Enterobacteriaceae*	46	38	7	1	84.4	70.9–92.6	100	98.6–100
MDR *Acinetobacter*	3	3	0	0	100	47.0–100	100	98.8–100
Carbapenem-resistant *Psuedomonas*	2	0	2	0	0	0.0–63.1	100	98.8–100
**Total**	**98**	**72**	**24**	**2**	**75.0**	**65.4–82.6**	**100**	**98.9–100**

MRSA: Methicillin resistant *Staphylococcus aureus*; MRSE: Methicillin resistant *Staphylococcus epidermidis;* VRE: Vancomycin resistant Enterococcus; ESBL: Extended spectrum beta-lactamase; MDR *Acinetobacter*: Multidrug resisant *Acinetobacter*

^a^ 95% confidence interval was calculated using Adjusted Wald Method (http://www.measuringu.com/wald.htm)

As confirmed by the combined disk method, a total of 46 extended-spectrum β-lactamase (ESBL) producing Gram negative bacteria were obtained, in which 97.8% (45/46) had valid Verigene results. Of these, 84.4% (38/45) were positive for *bla*_*CTX-M*_ in BC-GN test (Table 5). In the seven ESBL producers that were missed by the BC-GN test, *bla*_*CTX-M*_ gene were positively detected by multiplex PCR assay. These isolates includes *E*. *coli* (n = 3), *Enterobacter xiangfangensis* (n = 2), *K*. *pneumoniae* (n = 1), and *K*. *variicola* (n = 1) harbouring *bla*_*CTX-M-14*_ or *bla*_*CTX-M-15*_ as well as other β-lactamase genes such as *bla*_*SHV*_ and *bla*_*TEM*_ genes (Table 6). To evaluate the clinical impact of *bla*_*CTX-M*_ gene detection by the BC-GN assay, we calculated the positive predictive value (PPV) and negative predictive value (NPV) in predicting cefotaxime resistance in *Enterobacteriaceae* when using disk diffusion test results as the gold standard comparator. All 38 isolates positive for *bla*_*CTX-M*_ gene in BC-GN test showed a cefotaxime resistance phenotype. Among 175 Enterobacteriaceae detected by the BC-GN assay but negative for *bla*_*CTX-M*_ gene, 22 isolates were resistant to cefotaxime. Therefore, the PPV for cefotaxime resistance phenotype in these species by the BC-GN assay was 100% (38/38), whereas the NPV was 87.4% (153/175). The resistance genes harboured by the cefotaxime resistant Enterobacteriaceae isolates were characterized and were presented in Table 6.

**Table 6:** Resistance gene profile in Gram negative organisms collected in this study.

			Verigene BC-GN result	
Organisms	Resistance Gene profile	No. of isolates	Organisms	Resistance target
**Drug resistant Gram negative isolates detected by Verigene Test (n = 41)**				
*MDR A*. *baumannii*	OXA-23, OXA-51	3	*Acinetobacter spp*.	OXA
ESBL producing *E*. *coli*	CTXM-14	20	*E*.*coli*	CTXM
ESBL producing *E*. *coli*	CTXM-14, TEM	5	*E*.*coli*	CTXM
ESBL producing *E*.*coli*	CTXM-14, TEM, CIT	2	*E*.*coli*	CTXM
ESBL producing *E*.*coli*	CTXM-15	1	*E*.*coli*	CTXM
ESBL producing *E*. *coli*	CTXM-27	4	*E*.*coli*	CTXM
ESBL producing *E*.*coli*	CTXM-65	2	*E*.*coli*	CTXM
ESBL producing *K*. *pneumoniae*	CTXM-14, TEM	2	*K*. *pneumoniae*	CTXM
ESBL producing *K*.*variicola* ^*a*^	CTXM-14	1	Not Detected	CTXM
ESBL producing *K*.*variicola* ^*a*^	CTXM-65	1	Not Detected	CTXM
**Drug resistant Gram negative isolates failed to be detected by Verigene Test (n = 25)**				
ESBL producing *E*. *coli*	CTXM-14	1	*E*. *coli*	Not Detected
ESBL producing *E*. *coli*	CTXM-14, TEM	1	*E*. *coli*	Not Detected
ESBL producing *E*. *coli*	CTXM-14, SHV	1	*E*. *coli*	Not Detected
ESBL producing *E*. *coli*	CTXM-15, TEM	1	*E*. *coli*	Not Detected
ESBL producing *K*. *pneumoniae*	CTXM-15, TEM, SHV	1	*K*. *pneumoniae*	Not Detected
ESBL producing *K*.*variicola* ^*a*^	CTXM-14, DHA	1	Not Detected	Not Detected
ESBL producing *E*. *xiangfangensis* ^*a*^	CTXM-14, TEM	1	*Enterobacter spp*.	Not Detected
ESBL producing *E*. *xiangfangensis* ^*a*^	CTXM-15, TEM	1	No Call	No Call
Cefotaxime resistant *E*. *coli*	SHV	3	*E*. *coli*	Not Detected
Cefotaxime resistant *E*. *coli*	TEM	1	*E*. *coli*	Not Detected
Cefotaxime resistant *E*. *coli*	CIT	1	*E*. *coli*	Not Detected
Cefotaxime resistant *E*. *coli*	TEM, SHV	1	*E*. *coli*	Not Detected
Cefotaxime resistant *K*. *pneumoniae*	SHV	4	*K*. *pneumoniae*	Not Detected
Cefotaxime resistant *K*. *pneumoniae*	SHV, DHA	1	*K*. *pneumoniae*	Not Detected
Cefotaxime resistant *C*. *freundii* ^*a*^	Not Detected	2	*Citrobacter spp*.	Not Detected
Cefotaxime resistant *S*. *marcescens*	Not Detected	1	*S*. *marcescens*	Not Detected
Cefotaxime resistant *M*. *morganii* ^*a*^	DHA	1	Not Detected	Not Detected
Carbapenem-resistant *P*. *aeruginosa*	MOX	2	*P*. *aeruginosa*	Not Detected

^a^ species identified by 16s Sequencing

There were 5 isolates (3 *Acinetobacter spp*. and 2 *P*. *aeruginosa*) resistant to imipenem as determined by the disk diffusion test (Table 5). All the three *Acinetobacter spp*. were *Acinetobacter baumannii* with multidrug resistance phenotype. The BC-GN assay correctly detected the *bla*_*OXA*_ gene for all these isolates, and multiplex PCR further confirmed that they harboured *bla*_*OXA-23*_ *and bla*_*OXA-51*_ (Table 6). Unfortunately, no resistance gene was identified for the two imipenem resistant *P*. *aeruginosa* by the BC-GN assay. Multiplex PCR only revealed *bla*_*MOX*_ gene in both isolates (Table 6). To evaluate the clinical impact of *bla*_*OXA*_ gene detection, we calculated the PPV and NPV in detection of carbapenem resistance among *Acinetobacter spp*. (n = 4) and *P*. *aeruginosa* (n = 13). All the three isolates harbouring *bla*_*OXA*_ gene showed carbapenem resistant phenotype. Among the 14 isolates negative for *bla*_*OXA*_ gene, two showed resistant to carbapenem, giving the PPV and NPV for carbapenem resistance of 100% (3/3) and 85.7% (12/14), respectively.

### Time to Identification

The difference in time to report final bacterial identification using the Verigene Test and conventional culture-based method was investigated for 125 bacterial isolates (38 Gram positive and 87 Gram negative organisms) (Table 7). Of these, 23 belonged to *Staphylococcus* genus. The BC-GP reported bacterial identification and drug susceptibility result for cultures containing MSSA (n = 7), CoNS (n = 8), and MRSA (n = 10) an average of 46.65 h, 54.84, and even 91.10 h earlier than conventional culture-based method respectively. Among 13 positive cultures with *Streptococcus spp*., the final identification using culture methods were reported an average of 61.51 h after availability of the BC-GP results. For *Enterococci*, BC-GP results were available an average of 76.15 h before routine culture results. Importantly, this includes the identification of VRE, which was reported by BC-GP assay 99.15 h before the availability of final results using conventional culture and susceptibility testing.

**Table 7:** Difference in time to result between conventional culture-based method and the Verigene Test.

				Δ Time to Result^a^		
Organisms	No. of Isolates^b^	Average Time to identification by Culture-Based Method (h)	Average Time to Result by Verigene Test (h)	Average (h)	Range (h)	*p*-value
*Staphylococcus spp*.	23	63.76	2.35	61.41	22.65–494.65	*p*<0.001
MSSA	7	49	2.35	46.65	22.65–79.65	*p* = 0.012
MRSA	10	93.45	2.35	91.1	23.65–494.65	*p*<0.001
CNS	8	57.19	2.35	54.84	29.65–97.65	*p*<0.001
*Streptococcus spp*.	13	63.86	2.35	61.51	22.65–128.15	*p*<0.001
*S*. *pneumoniae*	3	42.83	2.35	40.48	22.65–56.65	p = 0.017
β-haemolytic Strept.	4	71.62	2.35	69.27	33.65–128.15	*p*<0.001
Viridians Group	6	55.08	2.35	52.73	25.65–83.65	*p*<0.001
*Enterococcus spp*.	2	78.5	2.35	76.15	53.15–99.15	*p*<0.001
VSE	1	55.5	2.35	53.15	n/a	n/a
VRE	1	101.5	2.35	99.15	n/a	n/a
Enterobacteriaceae	79	45.98	1.88	44.1	18.12–104.12	*P*<0.001
*P*. *aeruoginosa*	7	52.51	1.88	50.63	28.62–94.62	*p*<0.001
*Acinetobacter spp*.	1	76	1.88	74.12	n/a	n/a

^a^ Difference in time between BC-GP or BC-GN result and final culture-based identification and drug susceptibility test results

^b^ Only the isolates with concordant results from cultured-based method and the Verigene test were included for the time to result assessment.

The time required for identification for Gram negative organisms using the BC-GN assay was 1.88 h, which allowed the laboratory to report *Enterobacteriaceae* (n = 79) an average of 44.1 h earlier when compared with conventional culture-based method. Results for cultures containing other Gram negative organisms, including seven *P*. *aeruginosa* and one *Acinetobacter spp*., were available an average of 50.63 h and 74.12 h respectively before final identifications were reported using conventional methods (Table 7).

## Discussion

The major advantage of the Verigene Blood Culture Test is the rapid turnaround time, which allows same day analysis and reporting of bacterial identification and resistance determinants from positive blood cultures, and therefore facilitates both selection of appropriate antimicrobial treatment and implementation of infection control measures. The diagnostic accuracy of the assay has been shown to be promising in several studies conducted in other countries [14–17, 19, 20, 32–35]. However, the performance of molecular assays, particularly those relying on oligonucleotide probes to capture target organisms or resistance determinants, were subjected to genetic variation among bacterial lineages circulating in different geographic regions. Our previous study reported that a hybridization probe-based real time PCR assay designated for the detection of MRSA from nasal swabs, which demonstrated an overall sensitivity of 95.2% in a multicenter evaluation performed in U.S. [36], failed to detect almost 50% of MRSA isolates collected in Hong Kong [28]. Results indicated that the performance reported in one geographic area might not be reproducible in another, and local evaluation is necessary to reflect the applicability of a diagnostic assay in a particular region.

Among 364 blood cultures collected in this study, only 20 (5.5%) contained organisms not included in the Verigene test panel, illustrating that the capture probe targets of the Verigene test encompass the majority of organisms associated with bacteraemia cases in Hong Kong. However, when compared with other studies performed in the U.S., Europe and other Asian countries [14–17, 20, 32–35], the agreement in bacterial identification between the Verigene test and reference methods was relatively low in our study (86.8% vs 92.6% -98.6%) although the final call rate achieved 96.2%.

Additionally, as recognized as a limitation by the manufacturer in the CE-IVD version of the Verigene test that cultures containing more than one organism are prone to have false negative results, only 55.6% of polymicrobial cultures showed fully concordant results although 93.8% of them had the correct identification for at least one organism. The higher incidence of false negativity could be due to minor populations of the polymicrobial culture being present in a quantity lower than the limit of detection of the Verigene test or signal interference among multiple capture probes on the array slide. In this study, QMH showed the lowest overall agreement between the Verigene test and culture-based method among the four study sites, which was considered to be attributed to the relatively high proportion of polymicrobial cultures. Furthermore, polymicrobial cultures accounted for almost 10% of all collected positive blood cultures. The rate was relatively high in comparison to those observed in other recent evaluation studies of the BC-GP and BC-GN assay [14, 17, 19, 27, 32, 34]. This might also contribute to the low overall concordance obtained in present study.

With respect to Gram positive organisms, *Staphylococcus spp*. are the most frequently Gram positive bacteria isolated from blood cultures in this study. With the exception of one *S*. *aureus* culture reported as “No Call”, the BC-GP assay accurately detected all *S*. *aureus* and *S*. *epidermidis* isolates with 100% specificity. Identification and discrimination of MRSA and MSSA from other CoNS in positive blood culture shortly after broth positivity is of particular importance as it can greatly reduce health care cost, shorten time to appropriate antibiotic treatment, and improve patient outcome [37].

Similarly, for *Streptococcus spp*., those important causative organisms in BSI (*S*. *pyogenes*, *S*. *agalactiae*, *S*. *anginosus* Gp. and *S*. *pneumoniae*) were all correctly detected by the BC-GP assay. No false positivity was obtained for all species targets of *Streptococci* except *S*. *pneumoniae*. Previous studies showed that the most common misidentification by the BC-GP assay were attributed to *S*. *mitis* group incorrectly identified as *S*. *pneumoniae* due to high sequence similarity [14, 16, 19]. However, the five *S*. *mitis* groups isolated in this study were either correctly reported as “*Streptococcus spp*.” (n = 4) or given an invalid result “No Call” (n = 1). Instead, the BC-GP assay misidentified one *Kocuria koreensis* isolate as *S*. *pneumoniae* in the present study although the two species shared only 78% homology for 16s ribosomal RNA sequence (Data not shown). It is unclear whether this represented the presence of non-viable *S*. *pneumoniae* or contamination of this sample with *S*. *pneumoniae* nucleic acid.

In this study, all the false negative results reported by the BC-GP assay were attributed to *Enterococcus spp*., which represented the third most commonly isolated Gram positive genus. *E*. *faecalis* is more commonly isolated from blood cultures and is more susceptible to antibiotics when compared with *E*. *faecium*. A previous study indicated that 9.6% *E*. *faecium* isolates were resistant to vancomycin whereas vancomycin resistance was only found in 0.5% *E*. *faecalis* isolates [38]. This underscores the importance of identifying and differentiating these two organisms along with *vanA* and *vanB* gene detection in order to prescribe appropriate antimicrobial treatment for the patients with enterococcal bacteraemia. Unfortunately, we detected only 66.7% *E*. *faecalis* and 50% *E*. *faecium* isolates in contrast to 92.3%-100% *Enterococcus spp*. being correctly detected using the BC-GP assay in other studies [14–16, 19, 39]. Molecular epidemiological studies indicated that the major lineage of hospital-derived *E*. *faceium* isolates in China belonged to ST78 whereas ST17 was found to be the predominant strain in healthcare settings of the Western countries [40, 41]. We speculate that the lineage specific single nucleotide polymorphism may affect the binding affinity between the capture probes on the array and the target DNA from the organisms, and thus contribute to the false negative results, though this hypothesis was not tested.

The overall concordance rate of the BC-GN assay for the nine bacteria being identified was 86.4%. Interestingly, only 40.6% (13/32) discrepancies were accounted by polymicrobial cultures. The majority of mismatches indeed happened on the monomicrobial cultures. Blood cultures containing *K*. *pneumoniae* contributed to the largest number of false negative results (n = 14) reported by the BC-GN assay. Upon 16s rRNA and *yggE* gene sequencing, we found that 87.5% (7/8) monomicrobial cultures identified as *K*. *pneumoniae* by Vitek 2 but not detected by the BC-GN assay were actually *K*. *variicola*. The proportion was even higher than those reported by previous studies where 5.6% to 13.9% of *K*. *pneumoniae* that were not detected by the BC-GN assay were finally identified as *K*. *variicola* [27, 32, 35]. There have been an increasing number of reports indicating that *K*. *variicola* accounts for almost 10% *Klebsiella spp*. isolated obtained from clinical specimens and it has been associated with severe infections in humans including fatal sepsis [42–44]. Although literature revealed that *K*. *variicola* isolates were generally more susceptible to piperacillin and cephalosporins than *K*. *pneumoniae* [42], one ESBL (*bla*_*CTX-M-14*_) producing *K*. *variicola* isolate was obtained in this study. Of the seven *K*. *variicola* isolates, 57.1% (4/7) were obtained from QMH, which resulted in lower overall concordant rate when compared to the other three study sites. Given the clinical significance, the manufacturer should consider to include specific capture probes for *K*. *variicola* in the next generation of the assay. In addition to the discrepancy on *K*. *pneumoniae*, monomicrobal cultures of three *K*. *oxytoca*, two *E*. *coli* and two *P*. *aeruginosa* also failed to be detected by the BC-GN assay in spite of a single retest for each of these samples. The reason for the false negativity was unclear although we assumed that it might be due to the lineage of the bacteria not being recognized by capture probes or bacterial concentrations at the time of blood culture positivity below the limit of detection of the BC-GN assay [45, 46].

On the other hand, false positive results were reported for three monomicrobial and one polymicrobial culture. One blood culture broth tested positive as *K*. *oxytoca* by the BC-GN assay was subsequently identified as *R*. *planticola* by Vitek 2 and 16s rRNA sequencing. Because of the high phenotypic and genotypic similarity between these two organisms, the manufacturer listed the presence of *Raoultella spp*. in blood cultures as a potential risk of false positivity due to the cross-reactivity of the *K*. *oxytoca* capture probe. For the rest of misidentified cases, cross-reactivity of the capture probes is also considered as the major cause although the presence of non-viable organisms or nucleic acid contamination cannot be ruled out.

A total of 24 isolates that were not included in the Verigene test panel were obtained in this study. Most, if not all, of the Gram positive nontarget organisms, such as *Bacillus spp*. and *Corynebacterium spp*. are common normal skin flora and are generally considered as contaminants of blood cultures [47]. In contrast, most of the Gram negative nontarget organisms isolated in this study are associated with serious life threatening diseases. In line with an evaluation study of the BC-GN assay performed in Korea, *Aeromonas spp*. accounted for almost 5% of Gram negative organisms isolated from positive blood cultures [35]. In addition to being ubiquitous in aquatic environments, *Aeromonas spp*. can also cause various kinds of fatal and invasive infections [48]. Increasing cases with *Aeromonas* associated bacteraemia have been reported in healthcare settings in Asian countries [49]. Hence, we consider the exclusion of *Aeromonas* species and some other frequently encountered Gram negative pathogens, such as *M*. *morganii*, and *Salmonella spp*., from the panel as a potential pitfall of the use of the BC-GN assay in Asia.

For detection of drug resistance genes in Gram positive organisms, the BC-GP assay is highly accurate, with correct detection of *mecA* gene and *vanA* in all MRSA and VRE isolates respectively in this study. The negative percent agreement on drug resistance determination between the BC-GP assay and phenotypic methods was 100%, suggesting that therapy de-escalation to narrower antimicrobial agent based on the BC-GP assay result could proceed safely. In respect to identification of drug resistant Gram negative organisms, a major advantage of the Verigene test over other multiplex platforms, such as FilmArray, is that the BC-GN panel encompasses 6 genetic markers associated with resistance to various classes of β-lactam antibiotics. This allows us to timely and accurately detect Gram negative bacteria producing ESBLs and carbapenemases, which are of particular clinical concern as the therapeutic options for treating infections with these organisms are limited [50]. Nowadays, the vast majority of ESBLs belong to the CTX-M type enzyme. In this study, all of ESBL producers harboured *bla*_*CTXM*_. However, the BC-GN assay only identified 84.4% (38/45) of the *bla*_*CTXM*_ genes. Given that more than 80 types of CTX-M enzymes have been identified, the presence of rare types of CTX-M enzymes was initially speculated as the reason for false negative results. However, this was subsequently proven to be unlikely as bi-directional sequencing of the resistance gene confirmed that all the false-negative samples actually harboured the same CTX-M genotypes (i.e. *bla*_*CTX-M-14*_ or *bla*_*CTX-M-15*_) as those which were successfully detected (Table 6). Copy numbers of the plasmid below the detection limit could be another possible cause of false negativity for these cases, though the hypothesis has yet to be confirmed. In addition to *bla*_*CTX-M*_, other β-lactamase genes, such as *bla*_*SHV*_ *and bla*_TEM,_ can also transfer resistance to cefotaxime. We calculated the positive and negative predictability of CTX-M detection by the BC-GN assay for cefotaxime resistance, and the result was 100% and 87.4% respectively. This indicates that the use of penicillin and cephalosporin is not recommended whenever the *bla*_*CTX-M*_ gene is tested positive by the BC-GN assay, whereas the absence of detection of the genetic markers cannot be interpreted as an indication of susceptible isolates. The same interpretation can also be used for carbapenem resistance determination in Gram negative bacteria using the BC-GN assay. We determined 100% PPV for carbapenemase producing *A*. *baumannii*, however none of the carbapenem resistant *P*. *aeruginosa* isolates were tested positive for any resistance markers by BC-GN assay. The low NPV for carbapenem resistance in *P*. *aeruginosa* could be attributed to the fact that resistance mechanisms other than β-lactamase production, particularly active drug efflux pump, are more prevalent in *P. aeruginosa [51, 52]*.

As expected, the implementation of the Verigene test can greatly improve turnaround time for bacterial identification, and our results supported recent studies that reported a range of 30.5 to 127 h earlier than routine identification methods [14, 16]. In particular, the Verigene test showed the ability to differentiate the pathogenic Staphylococci and Streptococci from common blood culture contaminants, such as CoNS and Viridans Streptococci, an average of 61 h earlier than identification based on culture-based method with the sensitivities of 97.1%-100%. While antibiotic modification can be made based on organism identification alone for some bacteria, the selection of most appropriate antimicrobial therapy for certain bacterial pathogens, such as S. *aureus* and *Enterococcus spp*, greatly rely on the drug susceptibility results. Detection of *mecA* gene and *vanA*/*vanB* gene has been considered as confirmatory test for identification of MRSA and VRE. In this study, the Verigene platform enabled identification of MRSA and VRE to be completed 91 h to 99 h earlier as compared to conventional method. The rapid time to identification of MRSA and VRE facilitate earlier, tailor-made, modification to empiric therapy, which can result in better patient outcome. However, as discussed above, although the Verigene test can also be used to predict the susceptibility of Gram negative bacteria to β-lactam drugs, negative result for resistance markers cannot exclude β-lactamase-producing bacteria, and conventional drug susceptibility testing would be still required for these isolates. Direct comparison of the processing time of the BC-GN assay and conventional methods did not indicate the time saved for identification of β-lactamase-producing bacteria, and therefore only the time difference in bacterial identification but not drug susceptibility determination was reported for Gram negative bacteria in this study.

There were two potential weaknesses in the design of this study. First, the sample size was very low or even null for some specific targets, such as *S*. *lugdunensis* and resistance markers like *bla*_*IMP*_, *bla*_*VIM*_, *bla*_*KPC*_ and *bla*_*NDM*_, which are extremely rare in BSI cases within our region. Other studies tried to demonstrate the ability of Verigene test to identify these targets by using stimulated cultures. However, we observed that the accuracy of the Verigene test in stimulated cultures was always better than in clinical specimens [27, 32], and therefore did not truly reflect the performance of the assay in routine clinical practice. As we aimed to evaluate the applicability of the Verigene test in our clinical laboratories, only the prospectively collected blood cultures were tested although we understand that the diagnostic performance for some rarely encountered organisms and resistance markers could not be determined in this study. Second, since the Verigene test results were not used to modify the treatment regimen in our hospitals, we could not determine the impact of the Verigene test on the change in hospital stay, sepsis related mortality, cost of care, and the overall cost-effectiveness of the assay. A large-scale randomized controlled trial study is recommended to further investigate how the implementation of Verigene platform into routine workflow can benefit the patient outcome.

Compared to other studies which were subjected to a small sample size (<125 cultures) [17, 20, 32–34], a large proportion of simulated specimens (>60%) [32, 34], or a limited strain diversity based on the single centre-based design of the study [16, 17, 19, 20], the present study described the most comprehensive evaluation of combined use of the BC-GP and the BC-GN assay outside U.S. to date.

It should be noted that the time saved by the Verigene test might not be as pronounced as we described for the laboratories using MALDI-TOF MS. Recently, several studies demonstrated that short-term incubation of positive blood cultures on solid media prior to MALDI-TOF MS and Vitek 2 test can reduce the time to identification and the time to drug susceptibility result to 5 hour and 11–13 hour respectively [5, 6, 53]. Compared to the conventional culture-based test, these methods required shorter turnaround time without additional cost and workload. However, the increasing trends of resistance of Gram-negative bacteria to third-generation cephalosporins and to carbapenems as well as the high rates of methicillin-resistant Staphylococcus aureus across our region, further emphasize the need for rapid detection of resistance mechanism at a very early stage of infection given that the risk of death for septic patients increased by 10% for every hour of delay in administration of effective antibiotics [4]. In spite of high reagent cost, the Verigene test is a sample-to-result and fully automated system, which can report bacterial identification and clinically important resistance determinants from positive blood cultures in around 2 hour. Despite the limited clinical value of negative results for resistance marker, positive detection of *mecA*, *vanA* and other β-lactamase genes clearly indicate the presence of drug resistant bacteria, and modification of empirical treatment can be done at least 10 hour ahead of the availability of rapid drug susceptibility result by Vitek 2. To maximize the cost-effectiveness, it is suggested that the Verigene test should be constrained to patients with high emergency, such as those with severe sepsis and the immunocompromised patients, whereas the accelerated protocol using MALDI-TOF MS and Vitek 2 should be integrated in routine laboratory workflow to speed up the bacterial identification and of drug susceptibility test for all positive blood cultures.

In conclusion, the overall accuracy of the Verigene test were shown to be lower than other recent studies, which is mostly attributed to a relatively large number of polymicrobial cultures being tested in this study. The sensitivities and specificities were generally high for most of the common Gram positive and Gram negative organisms with the exception of *Enterococcus spp*. and *Klebsiella spp*. Detection of genetic markers of resistance by the assay reliably identified 100% MRSA, 100% VRE, 84.4% ESBL producers and 100% MDR-*Acinetobacter spp*. This can potentially facilitate earlier selection of appropriate antimicrobial therapy, improve infection control and reduce the total cost of care for patients infected with drug resistant organisms. Given the increasing prevalence of some nontarget organisms in BSI cases, such as *K*. *variicola* and *Aeromonas spp*., inclusion of capture probes for these organisms will greatly improve the applicability of the Verigene test in our region.
